# Screening for Multiple Types of Family Violence: Development and Validation of the Family Polyvictimization Screen

**DOI:** 10.3389/fpubh.2019.00282

**Published:** 2019-10-01

**Authors:** Ko Ling Chan, Qiqi Chen, Mengtong Chen, Camilla K. M. Lo, Lu Yu

**Affiliations:** Department of Applied Social Sciences, The Hong Kong Polytechnic University, Hong Kong, China

**Keywords:** family polyvictimization, screen, intimate partner violence, child abuse and neglect, elder abuse, validation

## Abstract

**Objective:** Different types of violence tend to co-occur within a family where the members often share common family characteristics, a situation described as family polyvictimization. In response to the lack of a validated screening tool, this study developed and validated the Family Polyvictimization Screen (FPS), the first brief screening tool applicable to members of the same family with up to three generations.

**Methods:** The FPS was designed to screen family polyvictimization by assessing and capturing different types of violence, including child abuse and neglect (CAN), intimate partner violence (IPV), and elder abuse. The FPS was compared with the Criterion Standard scales. It is suitable for use as a self-report for individual family members for specific violence or as a proxy report for an adult family member to serve as informant. In this study, a community sample of 445 households was recruited from Hong Kong (*n* = 250) and Shanghai (*n* = 195). One adult parent from each three-generation family was selected as the informant to report all family polyvictimization experiences in the preceding year.

**Results:** Moderate to high agreement (79.1–99.8%) was found between the FPS and the standard measurements, such as the revised Conflict Tactics Scales (CTS2) and the Conflict Tactics Scales: Parent-Child Version (CTSPC). Exceptions appeared in regard to physical assault on elders due to the rarity of reported cases. The specificity was high, while the sensitivity estimates appeared low, especially for the more sensitive sexual abuse cases.

**Conclusion:** The validated FPS demonstrated its potential utility as a brief tool for screening family polyvictimization in clinical settings with substantial agreement and satisfactory accuracy in the Chinese population.

## Introduction

Family polyvictimization is defined as the co-occurrence of child abuse and neglect (CAN), parental intimate partner violence (IPV), and elder abuse against different members in the same family (1). In past decades, studies have examined an array of different types of violence toward different family members either by focusing on one specific type or by covering various types and intensities of violence (2). Recent studies have confirmed that the effects of two or more types of violence victimization experiences could lead to more severe and less reversible impacts on the victims (1, 3). A recent study revealed that ~2.5% of families in China have experienced multiple types of violence (1), suggesting that family polyvictimization is not a rare phenomenon and highlighting the need for more research attention to be focused on this concept.

Currently, different types of family violence are often captured individually using different tools for specific targets or settings. For example, the common tools available for studying IPV include the Index of Spouse Abuse, the Danger Assessment Screen, and the revised Conflict Tactics Scales (CTS2). These tools have been widely used and modified to fit a wide range of practical uses or to address various research objectives and designs (4, 5). The usefulness of these measures has been promising; however, some of them comprise numerous detailed items that require a relatively long time to complete, thereby limiting their application as a quick screening tool for use in clinical settings. Some existing scales have been employed as Criterion Standard for screening or identifying IPV in clinical settings. For instance, the Chinese Abuse Assessment Screen (AAS) and the Hurt, Insult, Threaten, and Scream Scale (HITS) are brief assessments composed of three to four questions that have been proved reliable (6, 7). Yet, these brief screening tools have also been criticized as being unable to capture sufficient information; for example, the AAS focuses only on female victims, and it has been shown to be insensitive in capturing minor episodes of IPV (8). With the existing screening and assessment tools for IPV, striking a balance between speediness and thoroughness could be a challenging task.

Apart from the difficulty in balancing measuring speed and thoroughness, current screening or assessment tools may also face other types of challenges. For example, when assessing elder abuse, a relatively new issue in the field, researchers may face limitations in scope among the existing tools (9). The unique concerns of elderly people with regard to the administration of assessments have been recognized: for example, memory difficulties and visual impairments (10). To overcome these challenges, studies on elder abuse usually adopt structured face-to-face interviews with the elderly or their caregivers (11). However, little progress has been made in validating measures of sexual abuse and neglect toward the elderly (12), resulting in researchers experiencing difficulties in estimating reliable figures or conducting relevant studies on these issues.

Similar challenges appear in assessments on violence among the young population, who could be easily affected by people from a wide range of settings, such as their nuclear family, extended family, school, and neighborhood. Currently, some scales on CAN have been adapted from existing assessment tools capturing other types of family violence among the adult population [e.g., the Conflict Tactics Scales: Parent-Child version (CTSPC) (13)]. As the roles and impacts of parents and other family members are prominent during early childhood, assessments of CAN often include items to capture children's experience of witnessing parental conflict and sibling violence. For instance, the Juvenile Victimization Questionnaire (JVQ) provides a comprehensive assessment of conventional crime, child maltreatment, peer and sibling victimization, sexual victimization, and witnessing/indirect victimization (14). This highlights the importance of including items that can thoroughly capture all aspects of violence experiences across victims at different stages of development.

Recent research on the assessment of violence has focused on the measurement of different subtypes of violent behaviors that constitute polyvictimization (15). As outlined by the Centers for Disease Control and Prevention, the four major subtypes of victimization are physical violence, sexual violence, stalking, and psychological aggression (16). Studies have noted that the assessment of child exposure to IPV is often limited to physical violence between parents instead of including other types, such as psychological aggression (17). Failure to assess or the tendency to underreport sexual abuse appears to be common in current research findings, and this may lead to its prevalence being underestimated when making decisions on future actions (18). The evidence above reinforces the critical importance of comprehensive screening approaches for polyvictimization to detect polyvictims in advancing our understanding of the co-occurrence of family violence and identifying directions for its prevention (19).

In response to the lack of a validated screening tool, this study developed and validated the Family Polyvictimization Screen (FPS), the first brief screening tool to assess CAN, IPV, and elder abuse within a family, covering different types of violence across all family members up to three generations (i.e., grandparents, parents, and children).

## Methods

### Sample and Procedures

We followed the research design and analyses employed in the previous validation of the Chinese Abuse Assessment Screen (AAS) (7). The purpose of the study is not to report on prevalence or correlates of family polyvictimization. We employed a convenience sample of households from various communities in Shanghai and Hong Kong. The two chosen cities are relatively developed compared to other cities in China. Households in central and suburban districts were selected and included to maximize the diversity of participants with different socioeconomic characteristics. Eligible households were those with at least one child aged 18 years or younger living together with their parents and/or grandparents. One parent or caregiver from each of the sampled families was randomly selected as the informant to report the victimization experiences of all family members. If more than one child or one grandparent were eligible, the one with the most recent birthday was selected as the target of the study. No identifying information of the participants was recorded, and anonymity, privacy, and the right to refuse were ensured in all procedures.

Participation in the study was voluntary, and each participant gave their informed consent prior to the interviews. Informants were given the choice of completing either a printed or computer-assisted questionnaire, and the completed questionnaires were collected by trained interviewers. Confidentiality was guaranteed by assigning a sample code to each completed questionnaire instead of using the respondent's name. The printed questionnaires and signed consent forms were kept in a locked cabinet that was only accessible to the principal investigator. Respondents using the computer-assisted questionnaire were only identified by code numbers so that under no circumstances could the information be revealed outside of the research team. All the interviewers received intensive training on the procedure and ethical issues related to working with participants who report violence. All participants were given an information card with details about social services related to violence prevention to enable them to seek help whenever necessary. The Human Subjects Ethics Subcommittee of the authors' affiliated university provided ethics approval for the entire study.

### Measures

#### The Family Polyvictimization Screen

Items developed for the FPS were constructed by a group of medical and social sciences professionals led by the first author. The items were grouped into four modules: (a) IPV victimization, (b) IPV perpetration, (c) CAN, and (d) elder abuse. The 11 items of the four modules of the FPS and examples of the violent acts reported by respondents as a reference are listed in the Appendix. The IPV victimization and IPV perpetration modules both consisted of three items covering psychological aggression, physical assault, and sexual abuse. The CAN module comprised three items assessing psychological aggression, physical assault, and neglect of children, while the elder abuse module was composed of two items, namely, psychological aggression, and physical assault. We considered including sexual abuse, elder neglect, and financial exploitation. However, due to the lack of existing assessment tools for other forms of victimization, including elder neglect, elder sexual abuse, and financial exploitation, we could not validate those items using Criterion Standard in this study. Thus, we tested two items at this stage.

All 11 items were dichotomous questions. Sample items include “Have you ever experienced physical victimization?” and “Has your child ever been neglected?” Each question was accompanied with examples of the relevant violent behaviors to help the respondents assess and estimate whether they had witnessed or experienced such violence before. For example, psychological aggression could be the behaviors of a family member who “yells at you, is hypercritical of you, shames you, ridicules you, monitors you, isolates you from friends/family, threatens to hit you or throw something at you, accuses you, or destroys something that belongs to you.” These examples were referenced to the items listed in the Criterion Standard scales.

When respondents provided a “yes” response to a question, they were asked to indicate the identity of the perpetrator and the time frame of the victimization experiences. For the IPV victimization and IPV perpetration modules, the perpetrator(s) could be the respondents themselves, their partner, their father, their mother, their father-in-law, their mother-in-law, their child, or other relatives living in the household. For the CAN module, the possible perpetrator(s) were the same as those in the IPV modules, except for the “child” option, which was replaced by “sibling(s).” For elder abuse, possible perpetrator(s) covered the respondents themselves, their partner, the partner of the elderly victim, their child, and other relatives living together in the household. The participants were asked to report whether the victimization “happened in the preceding year” or “happened before the preceding year.”

### “Criterion Standard” Scales for Validation

The Criterion Standard scales include a series of questions to identify specific types of victimization for both screening in clinical settings and identifying victims in research which are expected to detect true positive cases or dismiss negative cases (7).

#### IPV Perpetration and Victimization

The revised CTS2 was used as the standard to validate the IPV victimization and perpetration modules (20). The CTS2 questionnaire is commonly adopted around the globe for measuring the prevalence, chronicity, and severity of spousal conflicts. This study adopted all eight questions from the Psychological Aggression subscale, 12 questions from the Physical Assault subscale, and six questions from the Injury subscale, as well as seven questions from the Sexual Coercion subscale for comparison.

#### Child Abuse and Neglect

In the case of CAN, we combined items from the CTSPC (13) and the JVQ (14), with the justification that neither scale on its own covered all the modules that we developed for validation. The psychological aggression and physical assault parts of the CAN module were compared with questions from the CTSPC, including the five questions from the Psychological Aggression subscale and 13 questions from the Physical Assault subscale. On the other hand, items related to child neglect were validated by those extracted from the JVQ, including five questions from the Supplemental Neglect subscale.

#### Elder Abuse and Neglect

The modified Conflict Tactics Scales (CTS) were used to validate the elder abuse module (20). This study employed 10 questions of the Psychological Aggression subscale and 13 questions of the Physical Assault subscale of the CTS.

For all the questions on victimization, the respondents were asked to indicate the time frame of the experiences: “happened in the preceding year,” “happened before the preceding year,” or “never happened.” When they provided a “yes” response to any of the victimization items, they were then asked to indicate specifically the perpetrator who conducted the specific violent behavior against the victim. The perpetrator options were the same as those in the items developed for the FPS.

#### Demographic Characteristics

A series of questions was used to collect information on the demographic, socioeconomic, and family characteristics of the respondents and their family members. Participants were asked to report family members' gender, age, residence status (i.e., whether they were living together in the same household), highest education level, employment status, marital status, and family income and whether they were receiving any social security assistance.

### Statistical Analyses

Demographic, socioeconomic, and family factors were summarized in descriptive statistics. Between-group comparisons were conducted to ensure there was no significant difference between the subsamples recruited from Hong Kong and Shanghai. The levels of agreement of the FPS items and those from the Criterion Standard were compared and analyzed using kappa coefficients. To assess the diagnostic accuracy and utility of using the FPS for screening various types of victimization, the sensitivity (SE), specificity (SP), positive and negative predictive values (PPV & NPV), and the positive and negative likelihood ratios (PLR & NLR) were computed using the related Criterion Standard (21). If the sum of sensitivity and specificity was >1, the FPS would be considered as a useful tool (22). In this study, blank answers were treated as missing values in the analyses. All estimates were accompanied by an exact 95% confidence interval, a *p*-value <0.05 was considered statistically significant, and all statistical analyses were performed in SPSS version 23.0.

## Results

Data from 445 parents and their families, 250 from Hong Kong, and 195 from Shanghai, were analyzed in this study, with response rates of 77.8 and 86.2%, respectively. The non-responses were mainly non-contacts, and <5% were refusals. No participants dropped out after agreeing to take part in the study. In the sample, 48.0% of the Hong Kong families had one child in the family and 44.8% had two, while the majority of the Shanghai families (83.1%) had only one child. The mean ages of parents were similar in the subsamples from the two cities, with the fathers having a mean age of around 40.0 years (SD = 9.52) and the mothers a mean age of around 38.5 years (SD = 7.17). The mean age of the selected children was 9.0 years (SD = 2.91), while the mean ages of the grandparents were 70 (Hong Kong) and 61 (Shanghai). Most of the parents were married or cohabiting with their current partner (95.5%) and lived together with their children (97.3%). Approximately 11.2% of the grandparents from Hong Kong and 26.5% of the grandparents from Shanghai lived with parents and children in the same household. The results showed no significant difference in demographic background between the subsamples from the two cities.

Table 1 shows the percentages of agreement and the kappa coefficients between the items from the FPS and the items from the selected Criterion Standard. Overall, moderate agreements were found between the FPS items and those from the standards, although the kappa coefficients ranged from fair (around 0.20) to substantial (over 0.70). For elderly physical assault, however, the kappa coefficients were not available, except for assault by elderly partner. Comparisons were not possible due to the limited number of cases (n ≤ 1) reported by the respondents.

**Table 1:** Agreement between the family polyvictimization scale and the criterion standards (*N* = 445).

**Type of abuse**	**Agreement % (95% CI)**	**Kappa coefficient**	***p*-value**
**IPV VICTIMIZATION (RESPONDENT)**			
Psychological aggression	87.0 (83.5–90.0)	0.737	<0.001
Physical assault/Injury	91.2 (88.2–93.7)	0.565	<0.001
Sexual abuse	97.8 (95.9–98.9)	0.490	<0.001
**IPV VICTIMIZATION (PARTNER)**			
Psychological aggression	87.0 (83.5–90.0)	0.729	<0.001
Physical assault/Injury	90.8 (87.7–93.3)	0.404	<0.001
Sexual abuse	97.8 (95.9–98.9)	0.158	<0.001
**CHILD ABUSE–PSYCHOLOGICAL AGGRESSION**			
By respondent	82.2 (78.4–85.7)	0.643	<0.001
By partner	79.1 (75.0–82.8)	0.579	<0.001
By grandparents	89.0 (85.7–91.7)	0.593	<0.001
By brothers and sisters	96.2 (94.0–97.8)	0.544	<0.001
**CHILD ABUSE–PHYSICAL ABUSE**			
By respondent	82.0 (78.1–85.5)	0.519	<0.001
By partner	82.7 (78.9–86.1)	0.382	<0.001
By grandparents	94.2 (91.6–96.2)	0.384	<0.001
By brothers and sisters	96.6 (94.5–98.1)	0.269	<0.001
**CHILD ABUSE–NEGLECT**			
By respondent	92.6 (89.7–94.8)	0.202	<0.001
By partner	95.3 (92.9–97.1)	0.378	<0.001
By grandparents	96.4 (94.2–97.9)	0.410	<0.001
**ELDERLY ABUSE–PSYCHOLOGICAL AGGRESSION**			
By respondent	93.7 (91.0–95.8)	0.529	<0.001
By partner	93.9 (91.3–96.0)	0.440	<0.001
By grandparents	91.7 (88.7–94.1)	0.368	<0.001
By respondents' children	96.2 (94.0–97.8)	0.302	<0.001
**ELDERLY ABUSE–PHYSICAL ASSAULT**			
By respondent	99.3 (98.0–99.9)	–	–
By partner	99.8 (98.8–100.0)	–	–
By grandparents	99.3 (98.0–99.9)	0.397	<0.001
BY respondents' children	99.8 (98.8–100.0)	–	–

*IPV, intimate partner violence*.

Table 2 shows the accuracy of the items on various types of victimization in the preceding year. The sensitivity estimates were generally satisfactory, ranging from 54.5 to 81.0%, except for child neglect, the sensitivity estimate for which was only 16.1%. The specificity estimates ranged from 83.8 to 99.8%.

**Table 2:** Accuracy of the family polyvictimization scale for screening preceding-year family victimization (*N* = 445).

**Polyvictimization scale**		**Gold standards**						
	**++ a− c**	**−bd**	**Se % (95% CI)**	**Sp % (95% CI)**	**PPV % (95% CI)**	**NPV % (95% CI)**	**PLR (95% CI)**	**NLR (95% CI)**
**IPV VICTIMIZATION (RESPONDENT)**								
Psychological aggression	166	12	78.3 (72.8–83.9)	94.8 (92.0–97.7)	93.3 (89.6–96.9)	82.8 (78.2–87.3)	15.2 (8.7–26.5)	0.23 (0.18–0.30)
	46	221						
Physical assault/Injury	31	14	55.4 (42.3–68.4)	96.4 (94.6–98.3)	68.9 (55.4–82.4)	93.8 (91.4–96.1)	15.4 (8.7–27.1)	0.46 (0.35–0.62)
	25	375						
Sexual abuse	5	1	35.7 (10.6–60.8)	99.8 (99.3–100.2)	83.3 (53.5–113.2)	97.9 (96.6–99.3)	–	0.64 (0.44–0.95)
	9	430						
**IPV VICTIMIZATION (PARTNER)**								
Psychological aggression	147	14	77.0 (71.0–82.9)	94.5 (91.7–97.3)	91.3 (87.0–95.7)	84.5 (80.3–88.7)	14.0 (8.3–23.4)	0.24 (0.18–0.32)
	44	240						
Physical assault/Injury	17	16	40.5 (25.6–55.3)	96.0 (94.1–97.9)	51.5 (34.5–68.6)	93.9 (91.6–96.2)	10.2 (5.6–18.7)	0.62 (0.48–0.80)
	25	387						
Sexual abuse	1	2	11.1 (0.0–31.6)	99.5 (98.9–100.2)	33.3 (0.0–86.7)	98.2 (96.9–99.4)	24.2 (2.4–243.5)	0.89 (0.71–1.13)
	8	434						
**CHILD ABUSE-PSYCHOLOGICAL AGGRESSION**								
By respondent	201	32	81.0 (76.2–85.9)	83.8 (78.6–88.9)	86.3 (81.8–90.7)	77.8 (72.2–83.4)	5.0 (3.6–6.9)	0.23 (0.17–0.30)
	47	165						
By partner	156	37	73.6 (67.7–79.5)	84.1 (79.4–88.8)	80.8 (75.3–86.4)	77.8 (72.6–82.9)	4.6 (3.4–6.3)	0.31 (0.25–0.40)
	56	196						
By grandparents	47	14	57.3 (46.6–68.0)	96.1 (94.2–98.1)	77.0 (66.5–87.6)	90.9 (88.0–93.8)	14.9 (8.6–25.7)	0.44 (0.35–0.57)
	35	349						
By brothers and sisters	11	10	61.1 (38.6–83.6)	97.7 (96.2–99.1)	52.4 (31.0–73.7)	98.3 (97.1–99.6)	26.1 (12.8–53.3)	0.40 (0.22–0.71)
	7	417						
**CHILD ABUSE-PHYSICAL ABUSE**								
By respondent	70	28	57.4 (48.6–66.2)	91.3 (88.3–94.4)	71.4 (62.5–80.4)	85.0 (81.3–88.8)	6.6 (4.5–9.7)	0.47 (0.38–0.58)
	52	295						
By partner	36	27	41.9 (31.4–52.3)	92.5 (89.8–95.2)	57.1 (44.9–69.4)	86.9 (83.5–90.3)	5.6 (3.6–8.6)	0.63 (0.52–0.75)
	50	332						
By grandparents	9	4	29.0 (13.1–45.0)	99.0 (98.1–100.0)	69.2 (44.1–94.3)	94.9 (92.8–97.0)	30.0 (9.8–92.1)	0.72 (0.57–0.90)
	22	410						
By brothers and sisters	3	10	37.5 (4.0–71.0)	97.7 (96.3–99.1)	23.1 (0.2–46.0)	98.8 (97.8–99.9)	16.4 (5.5–48.5)	0.64 (0.37–1.09)
	5	427						
**CHILD ABUSE-NEGLECT**								
By respondent	5	7	16.1 (3.2–29.1)	98.3 (97.1–99.6)	41.7 (13.8–69.6)	94.0 (91.8–96.2)	9.5 (3.2–28.3)	0.85 (0.73–1.0)
	26	407						
By partner	7	5	30.4 (11.6–49.2)	98.8 (97.8–99.8)	58.3 (30.4–86.2)	96.3 (94.5–98.1)	25.7 (8.8–74.8)	0.70 (0.54–0.92)
	16	417						
By grandparents	6	8	42.9 (16.9–68.8)	98.1 (96.9–99.4)	42.9 (16.9–68.8)	98.1 (96.9–99.4)	23.1 (9.2–57.6)	0.58 (0.37–0.92)
	8	423						
**ELDERLY ABUSE-PSYCHOLOGICAL AGGRESSION**								
By respondent	18	13	54.5 (37.6–71.5)	96.8 (95.2–98.5)	58.1 (40.7–75.4)	96.4 (94.6–98.2)	17.3 (9.3–32.1)	0.47 (0.32–0.68)
	15	399						
By partner	12	8	38.7 (21.6–55.9)	98.1 (96.7–99.4)	60.0 (38.5–81.5)	95.5 (93.6–97.5)	20.0 (8.9–45.3)	0.63 (0.47–0.83)
	19	406						
By grandparents	13	17	39.4 (22.7–56.1)	95.9 (94.0–97.8)	43.3 (25.6–61.1)	95.2 (93.1–97.2)	9.5 (5.1–17.9)	0.63 (0.48–0.83)
	20	395						
By respondents' children	4	12	44.4 (12.0–76.9)	97.2 (95.7–98.8)	25.0 (3.8–46.2)	98.8 (97.8–99.9)	16.1 (6.4–40.5)	0.57 (0.32–1.03)
	5	424						
**ELDERLY ABUSE-PHYSICAL ASSAULT**								
By respondent	0	2	–	–	–	–	–	–
	1	442						
By partner	1	0	–	–	–	–	–	–
	1	443						
By grandparents	1	1	33.3 (0.0–86.7)	99.8 (99.3–100.2)	50.0 (0.0–119.3)	99.5 (98.9–100.2)	147.3 (11.8–1847.0)	0.67 (0.30–1.49)
	2	441						
By respondents' children	1	0	–	–	–	–	–	–
	1	443						

*CI, confidence interval; +, positive response; −, negative response; a, true positive; b, false negative; c, false positive; d, true negative; IPV, intimate partner violence; NLR, negative likelihood ratio; NPV, negative predictive values; PLR, positive likelihood ratio; PPV, positive predictive values; Se, sensitivity; Sp, specificity*.

## Discussion

The findings from this study provide preliminary supportive evidence for the effectiveness of the 11-item FPS as a brief screening tool to identify cases involving family polyvictimization in the Chinese population. Moderate agreements were found between the FPS and the Criterion Standard. Concerning violent behaviors inflicted by the respondents themselves, the sensitivity estimates were generally satisfactory. From 54.5 to 81.0% cases were confirmed as positive by the Criterion Standard when the FPS reported positive. However, for some other types of violence, such as IPV sexual abuse and elderly abuse, the sensitivity estimates were around 30–40%, which were relatively low. Yet, all of the sensitivity estimates in this study were >“1–specificity,” reflecting that the FPS is an informative assessment tool (22). Specificity estimates indicated that when the response on the FPS was negative, it was likely that the response on the relevant standard assessments was also negative. The relatively satisfactory negative predictive values (77.8–99.5%) for the various forms of victimization suggested that among those who were screened negative by the FPS, the probability of victimization-free responses in the standards was substantially high. In contrast, the findings on the sensitivity estimates and the positive predictive values were mixed. The positive likelihood ratios provided further support for the usefulness of the FPS. In this study, most of the values were >10, reflecting a high increase in the probability of having experienced the specific violence given a positive response to the FPS. The relatively lower sensitivity of the FPS for IPV sexual abuse, elderly abuse, and child neglect may have been caused by the lower prevalence rates of these types of violence in this sample. Thus, the number of abuse cases identified may not have been sufficient to compute for high sensitivity estimates. A larger sample will be required to further test the sensitivity of the FPS for IPV sexual abuse and elderly abuse.

There are limitations of the study's design that may be caused by underreporting. In this study, adult parents were recruited as informants to report the victimization experiences of their three-generation family, including grandparents, parents (respondents), and children. Adult parents were expected to be the most familiar with the situations and experiences of other family members and thus the ones who could provide credible information about the details of the incidents and contexts of violence. In proxy reports, underreporting could be a concern, especially when the proxy is the perpetrator or when the proxy is not familiar with the reported target. The ideal arrangement is to involve all family members in reporting their own experience of victimization. However, this may involve other limitations: for example, interviewing children directly to gain information on retrospective traumatic experiences has been a controversial ethical topic in the field (23, 24), and some may argue that elderly people are basically less capable of responding to written questionnaires due to memory and visual impairments (10). Yet, in busy environments such as clinical settings, proxy reports on family polyvictimization by a single informant could be a possible solution. In fact, past studies have found moderate between-partner agreement on IPV perpetration and victimization and satisfactory parent-child agreement on minor child abuse such as corporal punishment (25–27), providing support for the use of proxy reports in the early-stage screening of family polyvictimization when individual self-reports are not feasible. The decision to invite parents to be informants might also be justified by the findings from past research suggesting that adult parents, as proxies, could provide generally adequate and comparable information to child self-reports about the experiences of children (1, 14). In view of the limitations that may be caused by engaging different family members in different cultures in reporting victimization, future studies may consider to test the FPS in different countries and to engage different informants to demonstrate the effectiveness and reliability.

Except in the case of assault by elder partner, accuracy estimates were not available in regard to physical aggression toward elderly people. The FPS item on psychological aggression demonstrated satisfactory accuracy, while the accuracy of the item on physical assault appeared somewhat lower. This provides the literature with divergent evidence demonstrating that elder physical abuse can be more readily measured than the more subtle psychological form Schofield et al. (12, 28). A possible explanation for this is that respondents might not realize that the identification of elder physical assault should be determined by the act itself rather than by the injury sustained from the act, which leads to a negative response with regard to physical abuse if no injury was observed. Besides, elder neglect and elder financial exploitation have no standardized scales for validation. Little progress has been made in validating measures of these types of violence, although recent efforts have expanded the understanding of elder abuse by covering financial exploitation or elderly self-neglect (29). Further research is needed to meet this challenge and to better capture the less examined types of elder victimization. Moreover, studies have revealed that caregivers as proxy might report extremely low rates of the elder violence and tend to recognize only severe observable symptoms (30). It is plausible to include elderly informants to reflect a more sensitive and real picture of victimization experiences.

### Implications

The development of the FPS has advanced the current screening assessments for violence by providing a brief tool covering several types of violence in the family for use in the Chinese context. This study demonstrated the FPS as a brief tool for use in detecting family polyvictimization. The development and validation of the FPS could be promising to facilitate future research on violence screening using a family-oriented approach, which in turn may promote proactive screening and better coordination of community responses for victims. It has been found that when one type of violence happens to a member of a family, the likelihood of revealing other types of violence to the same family member or to other family members will increase (1). Therefore, screening for family polyvictimization whenever one type of family violence is detected might be an effective way to detect and identify family polyvictimization early. A brief screening tool is key to extending current knowledge on family violence and the polyvictimization phenomenon (12).

To conclude, violent relationships often originate from a nuclear family and spill over into the extended family. The use of measures that assess only one or a few forms of victimization individually may impede our ability to understand some key aspects of family violence and polyvictimization, from identifying the potential polyvictims to examining the extent and comparing the relative effects of different types of victimization. The validated FPS has demonstrated its potential utility as a holistic tool for screening family polyvictimization in clinical settings with substantial agreement and satisfactory accuracy in the Chinese population.

## Data Availability Statement

The raw data supporting the conclusions of this manuscript will be made available by the authors, without undue reservation, to any qualified researcher.

## Ethics Statement

The studies involving human participants were reviewed and approved by the Human Subjects Ethics Sub-committee of the Hong Kong Polytechnic University (Reference Number: HSEARS20180706002). The patients/participants provided their written informed consent to participate in this study.

## Author Contributions

KC conceptualized the study design, interpreted the data, and critically revised the manuscript. KC and QC analyzed the data and drafted the manuscript. MC, CL, and LY interpreted the data and critically revised the manuscript. All authors approved the final manuscript as submitted.

### Conflict of Interest

The authors declare that the research was conducted in the absence of any commercial or financial relationships that could be construed as a potential conflict of interest.
