# Supplementary material for: Screening for Multiple Types of Family Violence: Development and Validation of the Family Polyvictimization Screen
Source: Front Public Health. 2019 Oct 1;7:282. doi: 10.3389/fpubh.2019.00282 (PMC6779820; doi:10.3389/fpubh.2019.00282)
Supplement: Supplementary file 1 [file Data_Sheet_1.docx]

**Appendix. Items of the Family Polyvictimization Scale**

|  | **IPV (respondent’s experience)** |
| --- | --- |
| 1. | Have you been psychologically hurt? |
| 2. | Have you been physically hurt? |
| 3. | Has anyone forced you to engage in sexual activities? |
|  | **IPV (partner’s experience)** |
| 4. | Has he/she been psychologically hurt by you? |
| 5. | Has he/she been physically hurt by you? |
| 6. | Have you forced him/her to engage in sexual activities? |
|  | **Child abuse** |
| 7. | Has he/she been psychologically hurt by anyone? |
| 8. | Has he/she been physically hurt by anyone? |
| 9. | Has he/she been neglected? |
|  | **Elderly abuse** |
| 10. | Has he/she been psychologically hurt by anyone? |
| 11. | Has he/she been physically hurt by anyone? |

Note. Examples provided for psychological hurt included yelling, being hypercritical, shaming, monitoring, isolating from others, threatening to hit, threatening to throw something, accusing, destroying belongings, etc. Examples provided for physical hurt included hitting or slapping, shaking, throwing something, pushing or shoving, grabbing, dragging hair, beating or kicking, choking, burning or scalding, injuring, etc. Examples provided for forced sexual activities included having sexual intercourse or touching private parts against one’s wishes, sexual harassment, ignoring one’s request to use a condom, taking nude photos against one’s will, etc. Examples provided for neglect included deprived of care and sufficient food, lacking a safe place to stay, etc. Examples provided for financial exploitation included money taken without one’s permission, being cheated or put under pressure to sign any financial documents, etc.
